# Fatty Liver Disease in Patients with Prediabetes and Overweight or Obesity

**DOI:** 10.3390/metabo13040531

**Published:** 2023-04-07

**Authors:** María Arias-Fernández, Sergio Fresneda, Manuela Abbate, Marina Torres-Carballo, Aina Huguet-Torres, Cristian Sánchez-Rodríguez, Miquel Bennasar-Veny, Aina M. Yañez, Carla Busquets-Cortés

**Affiliations:** 1 Department of Nursing and Physiotherapy, University of Balearic Islands, 07122 Palma, Spain; 2 Research Group on Global Health, University of Balearic Islands, 07122 Palma, Spain; 3 Research Group on Global Health and Lifestyle, Health Research Institute of the Balearic Islands (IdISBa), 07120 Palma, Spain; 4 ADEMA-SALUD Group of IUNICS, University of Balearic Islands, 07009 Palma, Spain; 5 Primary Care Research Unit of Mallorca, Public Health Service of the Balearic Islands (Ibsalut), 07003 Palma, Spain; 6 Sant Joan de Déu Hospital, 07007 Palma, Spain; 7 CIBER de Epidemiología y Salud Pública (CIBERESP), 28029 Madrid, Spain

**Keywords:** non-alcoholic fatty liver disease, prediabetic state, primary health care, fatty liver index, metabolic syndrome, chronic disease

## Abstract

Non-alcoholic fatty liver disease (NAFLD) is a global health problem associated with liver morbimortality, obesity, and type 2 diabetes mellitus. This study aimed to analyze the prevalence of NAFLD (defined as a fatty liver index [FLI] ≥ 60) and its association with other cardiovascular risk (CVR) factors in patients with prediabetes and overweight/obesity. The present cross-sectional analysis uses baseline data from an ongoing randomized clinical trial. Sociodemographic and anthropometric characteristics, CVR (assessed by the REGICOR-Framingham risk equation), metabolic syndrome (MetS), and FLI-defined NAFLD (cut-off value of ≥60) were assessed. The prevalence of FLI-defined NAFLD was 78% overall. Men exhibited a worse cardiometabolic profile as compared to women, specifically, with higher values of systolic blood pressure (137.02 ± 13.48 vs. 131.22 ± 14.77 mmHg), diastolic blood pressure (85.33 ± 9.27 vs. 82.3 ± 9.12 mmHg), aspartate aminotransferase (AST) (27.23 ± 12.15 vs. 21.23 ± 10.05 IU/L), alanine aminotransferase (ALT) (34.03 ± 23.31 vs. 21.73 ± 10.80 IU/L), and higher CVR (5.58 ± 3.16 vs. 3.60 ± 1.68). FLI-defined NAFLD was associated with elevated AST, ALT, and the presence of MetS (73.7%) and CVR for the whole sample. People with prediabetes present a high burden of comorbidities related to CVR, despite clinical follow-up, and it is recommended to actively begin working with them to reduce their risks.

## 1. Introduction

Non-alcoholic fatty liver disease (NAFLD) is one of the most common causes of liver disease [1]. It is characterized by the accumulation of fat in the hepatocytes, not related to excessive alcohol consumption [1,2]. The gold standard for the diagnosis of NAFLD is liver biopsy; however, this is an invasive procedure with associated risks and high cost [2]. The fatty liver index (FLI) has been shown to be a valid screening tool for NAFLD, showing efficiency and effectiveness [3].

NAFLD affected around 30% of the population between 1999 and 2019 [4]. Currently, NAFLD is expected to increase as a consequence of the rising prevalence of diabetes and obesity [4,5,6]. In Spain, the prevalence of NAFLD is between 25–30% [7,8,9].

The prevalence of NAFLD presents differently between sexes, with men having a higher prevalence than women, across all ages [9]. Nevertheless, in the case of postmenopausal or diabetic women of any age, their risk of NAFLD is higher compared to younger or metabolically healthy women [10].

NAFLD is a global health problem and is the main cause of liver-related complications and mortalities [2]. Moreover, NAFLD is closely linked to metabolic syndrome (MetS), obesity, and type 2 diabetes mellitus (T2D) and is a potential predictor of cardiovascular events [2,9,11,12,13,14]. In fact, NAFLD is the hepatic manifestation of MetS, with a 28% prevalence of MetS in NAFLD [13,14,15]. In addition, people with obesity exhibit a two-fold increase in NAFLD prevalence compared to people without obesity [8,16]. Likewise, the prevalence of NAFLD is further increased in T2D; in fact, 50–70% of people with T2D present NAFLD [5,8,17]. In turn, T2D is one of the most important risk factors for NAFLD-related complications such as non-alcoholic steatohepatitis or hepatocellular carcinoma [12,18,19].

In subjects with prediabetes, the prevalence of NAFLD varies between 40.7–55.7%, and it is associated with a worse cardiometabolic outcome [12,19,20,21,22]. Therefore, it is important to understand the epidemiology ofprediabetes and NAFLD to take preventive action [23,24,25]. Generally, studies have investigated each phenomenon separately, but in the present analysis, we aim to observe all comorbidities.

The mean objective of the present study was to analyze baseline data of an ongoing randomized clinical trial. The analyses aimed to evaluate the prevalence of FLI-defined NAFLD in a sample of patients with prediabetes and overweight or obesity and the association of FLI-defined NAFLD with cardiovascular risk (CVR).

## 2. Materials and Methods

### 2.1. Study Design

The present cross-sectional analyses use baseline data from the clinical trial “Effectiveness of a Nurse-led Personalized Telephone Intervention on Lifestyle Changes in Diabetes Prevention (PREDIPHONE)” (clinicaltrial.gov identifier: NCT04735640). The trial study methods have been detailed elsewhere [26]. Inclusion criteria for the PREDIPHONE trial were: age 25–75 years, BMI 27–40 kg/m^2^, and fasting plasma glucose (FPG) 100–125 mg/dL (prediabetes defined according to ADA criteria [26,27]). Exclusion criteria were: history of diagnosed diabetes (type 1 and 2), FPG ≥ 126 mg/dL, current treatment with antidiabetic medications, use of systemic glucocorticoids, having initiated lifestyle modification through diet and/or physical activity within the previous 3 months, history of any hematologic disease affecting HbA1c results, hospital admission or major surgery in the previous 3 months, pregnancy, terminal illness, institutionalization, dementia or cognitive impairment, presence of any medical or psychological condition limiting participation, or concomitant participation in another clinical trial.

The study followed the Declaration of Helsinki ethical standards, and all the procedures were approved by the Institutional Review Board of the Balearic Islands Health Service Research Ethics (CEI-IB Ref No: IB 3947/19 PI). All participants signed the informed consent.

Between May 2021 and September 2022, a total of 202 men and women (53.2% women) meeting the selection criteria were randomized and allocated to study groups. The trial was carried out across five primary healthcare centers in Majorca, Balearic Islands, Spain.

### 2.2. Demographic and Clinical Data Collection

Demographic and clinical data (medical history, use of medications, anthropometric characteristics, and blood and urine biochemical analyses) were collected during baseline visits by trained healthcare professionals.

According to the occupation declared, participants were assigned to either a higher social class, defined as “white collar” (executives, managers, university professionals, intermediate workers, and employees) or a lower social class, defined as “blue collar” (manual laborers), as suggested by the Spanish Society of Epidemiology [28].

Smoking behavior was recorded as never, former, or current smoker, according to the classification recommended by the World Health Organization (WHO) [29], used in multiple national surveys [30,31,32].

Anthropometric measurements, such as height, weight, BMI, and waist circumference (WC), were collected following the International Standards for Anthropometric Assessment (ISAK) [33]. Body weight and composition were measured by bioelectrical impedance (Tanita BC-418, Tanita Corp, Tokyo, Japan) [34]. Height was collected using a stadiometer, with the patient standing upright with the head in anatomical position (SECA 220 Seca 220 (CM) Telescopic Height Rod for Column Scales, Seca GmbH, Hamburg, Germany). WC was measured in duplicate using an anthropometric tape with the participant in a standing position; the mean of the two measures was used for analysis. WC was measured midway between the last rib and the top of the iliac crest. BMI was calculated according to standard formula which defines obesity as BMI ≥ 30 kg/m^2^ [35,36].

Blood pressure (BP) was measured in duplicate (1 min apart) in both arms, after a 15 min rest in a seated position, using a calibrated electric sphygmomanometer (OMRON M3, Healthcare Europe, Barcelona, Spain). The mean of the two measurements was calculated, and the arm with the highest BP was used for statistical analysis. BP was classified according to the 2018 European Society of Hypertension/European Society of Cardiology (ESC/ESH) criteria as normal (systolic BP (SBP) < 130, diastolic BP (DBP) < 85 mmHg), pre-hypertension (SBP 130–139 and/or DBP 85–89 mmHg), and hypertension (SBP ≥ 140, or DBP ≥ 90 mmHg, and/or using antihypertensive medications) [37].

Venous blood samples were taken after an overnight fast of 12 hours from the antecubital vein in suitable vacutainers without anticoagulant.

FPG, aspartate aminotransferase (AST), alanine aminotransferase (ALT), gamma-glutamyl transferase (GGT), total cholesterol, high-density lipoprotein cholesterol (HDL-C), and triglycerides (TG), were measured in serum on the Abbott ARCHITECT c16000 (Abbott Laboratories, Abbott Park, IL, USA), employing specific commercial kits. Low-density lipoprotein cholesterol (LDL-C) was calculated according to the Friedewald formula [38]. Hematological parameters were analyzed in whole blood in an automatic flow cytometer analyzer (Cell-Dyn Sapphire platform, Abbott Core Laboratory Systems, Lake Forest, IL, USA). Analyses were carried out at the Son Espases University Hospital, Palma, Majorca.

### 2.3. Definitions

#### 2.3.1. MetS

The International Diabetes Federation (IDF) defines MetS as the concomitant presence of central obesity expressed as WC ≥ 80 cm for females and ≥94 cm for males (central obesity can be assumed when BMI is >30 kg/m^2^ and WC is not measured) and any two of the following four factors: TG ≥ 150 mg/dL or specific treatment; HDL-C in females < 50 mg/dL and in males < 40 mg/dL or specific treatment; BP ≥ 130/85 mmHg or specific treatment; and FPG > 100 mg/dL or previously diagnosed T2D [39,40].

#### 2.3.2. Cardiovascular Risk

CVR at 10 years was estimated using the REGICOR-Framingham risk equation, validated in a Spanish adult population aged 35–74 years [41,42]. The equation expresses the risk as a percentage, and it uses variables associated with CVR such as sex, age, smoking, T2D, HDL-C, SBP, and DBP. Thus, we defined a percentage of <5.0% as indicating low risk, 5.0–9.9% as moderate risk, 10.0–14.9% as high risk, and >15.0% as severe risk.

The 10-year risk for fatal cardiovascular events was calculated using the SCORE equation, validated for subjects between 40 and 65 years, without diabetes [43]. A probability of 5% or above indicates high risk; thus, we divided subjects as having low risk (<0.99%), moderate risk (1–4.99%), high risk (5–9.99%), or severe risk (>10%).

#### 2.3.3. FLI as a Surrogate Measure of Fatty Liver

The FLI score is one of the most cost-effective and non-invasive indicators for the presence of NAFLD, and it is widely used in epidemiological studies [3,21,44].

The FLI was calculated using the formula developed by Bedogni et al. based on the measurements of TG, GGT, BMI, and WC [3]:

Fatty Liver Index (FLI) = (e^0.953 y)/((1 + e^0.953 y) × 100

where y = 0.953 × ln (TG) + 0.139 × BMI + 0.718 × ln (GGT) + 0.053 × WC − 15.745.

In the formula, TG is expressed as mg/dL, BMI as kg/m^2^, GGT as U/L, and WC in cm.

FLI can be divided into three scoring categories: FLI < 30, FLI 30–59, and FLI ≥ 60. Values of FLI below 30 exclude steatosis with a sensitivity of 87% and a specificity of 86%. FLI scores between 30 and 59 indicate undetermined risk; thus, fatty liver should be neither ruled in nor ruled out. Values above 60 determine the presence of steatosis, with a sensitivity of 61% and a specificity of 86% [3].

### 2.4. Statistical Analyses

Variables were tested for normality using the Kolmogorov–Smirnov test. Continuous variables, expressed as mean and standard deviation (±SD), were analyzed using the Student’s *t*-test, Pearson’s correlations, and analysis of variance (ANOVA), with post hoc evaluation using the Bonferroni contrast method. Categorical variables, expressed as counts and percentages (%), were compared by the Chi-square test (χ^2^) with post hoc evaluation by the Bonferroni method. Odds ratios (ORs) and corresponding 95% confidence intervals (CI) were calculated to evaluate the factors associated with FLI. Participants were categorized as FLI ≥ 60 and FLI < 60. Statistical analyses were carried out using the Statistical Package for the Social Sciences (SPSS) version 26.0 (IBM Company, New York, NY, USA). All statistical tests were two-sided, and *p*-values < 0.05 were considered statistically significant.

## 3. Results

### 3.1. General Characteristics of the Study Population

Of the 202 participants included in the PREDIPHONE study, 186 had available data on GGT and were included in the present analysis.

Of the 186 subjects, 97 were women (52.15%). The mean age was 59.26 ± 10.32 years. Sociodemographic and anthropometric characteristics, along with the biochemical parameters of the whole sample, stratified by sex, are shown in Table 1. Most individuals were blue-collar workers (78.5%), and presented obesity (73.7%) and dyslipidemia (57.5%); according to the IDF criteria, 73.7% presented MetS. A total of 69.4% of the participants were hypertensive. Of those, 24% were undiagnosed hypertensives, and 23% exhibited poor BP control, despite taking antihypertensive treatment. According to the REGICOR-Framingham risk equation, 33.3% of participants presented a moderate, and 5.4% presented a high, CVR. On the other hand, according to the SCORE equation, 51.6% of subjects had a moderate risk and 18.3% had a high risk of a fatal cardiovascular event at 10 years.

As for smoking habits, 18.6% of women and 11.2% of men were current smokers. As compared to women, men had statistically significantly higher values of SBP, DBP, AST, ALT, and a higher CVR, according to the REGICOR-Framingham risk equation, as well as lower HDL-C values.

### 3.2. Prevalence of FLI-Defined NAFLD

The prevalence of FLI-defined NAFLD was 78%, with a mean FLI value of 75.61.

Table 2 shows differences in anthropometric characteristics and biochemical parameters between FLI categories, also expressed as OR (95% CI). As compared to patients with FLI < 60, those with FLI ≥ 60 had significantly increased rates of obesity and MetS, higher BMI, and lower HDL-C values.

### 3.3. FLI-Defined NAFLD and CVR

As shown in Table 1, men had a higher prevalence of moderate and high CVR as compared to women, according to the Framingham-REGICOR risk equation. At the same time, the majority of women fell in the low-risk category for CVR. As for the SCORE equation, men were more likely to belong to the high-risk category and women to the low-risk category, as compared by sex.

As shown in Table 2, participants with FLI ≥ 60 presented a higher CVR according to the REGICOR-Framingham risk equation when compared to those with FLI < 60.

**Figure 1:**
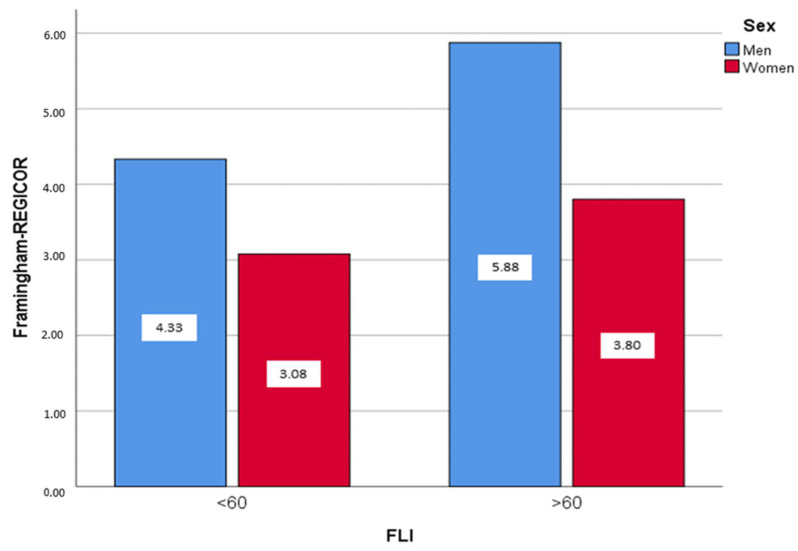
Mean Framingham-REGICOR values according to FLI categories (FLI < 60 and FLI ≥ 60) by sex. χ^2^ test, *p* < 0.05.

Figure 1 shows significant differences between FLI categories and sex for CVR. Participants with FLI ≥ 60 exhibited the highest CVR (5.88% men vs. 3.80% women) and those with FLI < 60 the least CVR (4.33% men vs. 3.08% women).

## 4. Discussion

The prevalence of FLI-defined NAFDL in a sample of patients with prediabetes and overweight/obesity included in the PREDIPHONE study was 78%, and higher in men (83.1%) than in women (73.2%). FLI-defined NAFLD was associated with a worse cardiometabolic profile and increased CVR.

The observed prevalence of FLI-defined NAFLD in our study population is higher than the prevalence of NAFLD observed in the general population (estimated at 30%) [4,11] and in individuals with overweight (43.64%) or obesity (56.71%) [45], but it is similar to that of subjects with prediabetes observed in previous studies (which ranges from 45% to 78%) and to that of patients with type 2 diabetes (55–70%) [22,46,47].

Our observations are in line with those of previous studies with a similar or younger population with prediabetes, describing high BMI, WC, TG, SBP, and DBP and lower values of HDL-C as possible risk factors contributing to NAFLD [8,47].

### 4.1. FLI-Defined NAFLD by Sex

Our findings of a difference in prevalence between sexes, with men experiencing a higher prevalence than women, is in accordance with the available evidence [9]. What is generally observed is a significant difference in prevalence between men and women of reproductive age. After menopause, the prevalence in women tends to increase, possibly due to the hormonal changes experienced during this phase, reducing the gap difference between sexes [9,48,49]. In our case, women had a mean age of 59.82 ± 10.24; thus, many of them were possibly menopausal. This could explain why, in our case, sex differences in prevalence are not as evident as in studies with younger women [9,10].

### 4.2. FLI-Defined NAFLD and MetS

The relationship between NAFLD and MetS has been reported in multiple studies [15,50,51].

Compared to previous studies [15,50,51], the prevalence of MetS in NAFDL in our study was higher. This could be explained by the study selectively including subjects with prediabetes and overweight/obesity, which, by definition, are at increased cardiometabolic risk.

### 4.3. FLI-Defined NAFLD and Associated Comorbidities

The relationship between CVR and NAFLD is extensively demonstrated [8,9,16,46]. Multiple studies have reported the impact of several cardiometabolic comorbidities, such as obesity, hypertension, and T2D, on NAFLD [8,14,52,53,54,55,56]. In our sample, NAFLD was associated with obesity and moderate CVR. We found no association between NAFLD and a high CVR, possibly due to the small number of subjects in this category.

Obesity and NAFLD show evidence of a complex relationship [8,16]. In our analysis, a significant relationship between obesity and NAFLD was observed, in agreement with other studies in the literature [8,16,45].

Hypertension and NAFLD exhibit a bidirectional relationship: while hypertension could aggravate liver disease, NAFLD could influence the development of hypertension [57]. Nonetheless, in this analysis, no differences in BP categories or values were observed between subjects, with and without NAFLD.

Several studies have shown that when NAFLD, or FLI-defined NAFLD, is concomitantly present with prediabetes, it increases the risk of progression to T2D [18,46].

### 4.4. Screening FLI-Defined NAFLD in Primary Health Care

Because the high prevalence of NAFLD in the prediabetic population is a risk factor for the development of T2D and cardiovascular disease, preventative actions should be considered [5,47]. In real-world practice, there is no screening or tracking of people affected by prediabetes and NAFLD [23,47,58]. In fact, only 25% of these individuals receive follow-ups and advice on eating habits, physical activity, or weight reduction [58]. Moreover, recent studies have highlighted the importance of screening and awareness of healthcare providers regarding prevention [23,24]. T2D prevention programs are implemented, although they could be extended to patients with prediabetes [19,24,59].

Routine NAFLD screening in high-risk population is cumbersome and not advised due to uncertainties surrounding diagnostic tests and treatment options [60]). Nevertheless, the FLI is a cost-effective, useful, and validated tool that could be used systematically in people with prediabetes to potentially provide early detection and control of comorbidities [61].

### 4.5. Study Limitations and Strengths

The present analysis has some limitations to be considered. First, the gold standard for the diagnosis of NAFLD is liver biopsy; however, due to its associated risks, invasiveness, and cost, it is unviable as a routine procedure. The FLI equation, on the other hand, serves as a useful alternative in identifying people at risk of NAFLD [46,61]. Secondly, the cross-sectional design of this analysis limits drawing conclusions on causality. Finally, the sample size is small, and the results may not be extrapolated to the overall prediabetic population.

The main strength of our study is that its baseline data belong to a randomized controlled trial, and its collection followed quality research standards.

## 5. Conclusions

Our results showed a much higher prevalence of FLI-defined NAFLD in our participants with prediabetes and overweight/obesity than in the general population. Furthermore, FLI-defined NAFLD was associated with worse anthropometric and biochemical parameters, hypertension, and CVR.

## Figures and Tables

**Table 1:** Anthropometric characteristics and biochemical parameters of the study population, overall and according to sex.

Variable	All *n* = 186	Men *n* = 89 (47.84%)	Women *n* = 97 (52.15%)	*p*-Value *
Age (years)	59.26 (10.32)	58.65 (10.43)	59.82 (10.24)	0.440
Social class				0.960
White collar	40 (21.5)	19 (21.3)	21 (21.6)	
Blue collar	146 (78.5)	70 (78.7)	76 (78.4)	
Smoking status				<0.001
Never	84 (45.2)	27 (30.3)	57 (58.8)	
Former	74 (39.8)	52 (58.4)	22 (22.7)	
Current	28 (15.1)	10 (11.2)	18 (18.6)	
BMI (kg/m^2^)	32.29 (3.53)	32.00 (3.32)	32.56 (3.71)	0.279
BMI categories				0.395
Overweight	49 (26.3)	26 (29.2)	22 (23.7)	
Obese	137 (73.7)	63 (33.9)	74 (76.3)	
WC (cm)	105.62 (10.19)	109.10 (8.90)	102.44 (10.29)	<0.001
SBP (mmHg)	133.98 (14.13)	137.02 (13.48)	131.22 (14.77)	0.005
DBP (mmHg)	83.76 (9.29)	85.33 (9.27)	82.35 (9.12)	0.029
BP categories				0.660
Normal	32 (17.2)	13 (14.6)	19 (19.6)	
Prehypertension	25 (13.4)	12 (13.5)	13 (13.4)	
Hypertension	129 (69.4)	64 (71.9)	65 (67.0)	
FPG (mg/dL)	108.76 (6.24)	109.44 (6.59)	108.13 (5.86)	0.155
HbA1c ^∇^	5.89 (0.32)	5.86 (0.33)	5.92 (0.32)	0.282
GGT (IU/L)	44.11 (60.06)	56.88 (82.51)	32.40 (20.53)	0.008
AST (IU/L) ^+^	24.11 (11.79)	27.23 (12.75)	21.23 (10.05)	0.001
ALT (IU/L) °	27.68 (18.95)	34.03 (23.31)	21.73 (10.80)	<0.001
Cholesterol (mg/dL)	198.28 (35.01)	194.73 (37.74)	201.54 (32.15)	0.186
HDL-C (mg/dL)	49.98 (12.20)	46.26 (9.91)	53.39 (13.13)	<0.001
LDL-C (mg/dL)	119.55 (29.74)	118.84 (30.59)	120.19 (29.11)	0.761
TG (mg/dL)	152.04 (143.21)	163.62 (195.67)	141.41 (64.73)	0.310
Presence of dyslipidemia	107 (57,5)	52 (58.4)	55 (56.7)	0.812
Presence of MetS	137 (73,7)	65 (73.0)	72 (74.2)	0.854
REGICOR	4.55 (2.68)	5.58 (3.16)	3.60 (1.68)	<0.001
Categories of Framingham-REGICOR				<0.001
Low risk ^a^	113 (60.8)	39 (43.8)	74 (76.3)	
Moderate risk ^a^	62 (33.3)	40 (44.9)	22 (22.7)	
High risk ^a^	11 (5.9)	10 (11.2)	1 (1.0)	
SCORE	2.91 (2.62)	3.69 (2.82)	2.20 (2.20)	<0.001
Categories of SCORE				<0.001
Low risk ^a^	52 (28.0)	15 (16.9)	37 (38.1)	
Moderate risk	96 (51.6)	47 (52.8)	49 (50.5)	
High risk ^a^	38 (20.4)	27 (30.3)	11 (11.3)	
FLI	75.61 (19.02)	79.26 (17.53)	72.27 (19.79)	0.012
FLI categories				0.102
<60	41 (22.0)	15 (16.9)	26 (26.8)	
≥60	145 (78.0)	74 (83.1)	71 (73.2)	

Data are expressed as mean (standard deviation) or count (percentage). * *p*-values for comparison between men and women, obtained by independent sample t-test for continuous variables or by Chi-square test for categorical variables. Post hoc test by Bonferroni method: ^a^ significant difference between men and women. ^+^ AST available for *n* = 179 (men, *n* = 86; women, *n* = 93); °ALT available for *n* = 182 (men, *n* = 88; women, *n* = 98); **^∇^** HbA1c available for *n* = 149 (men, *n* = 72; women, *n* = 77). Abbreviations and category definition: FLI, fatty liver index; BMI, body mass index; BMI categories: overweight (BMI 25 to 30 kg/m^2^); obese (BMI ≥ 30 kg/m^2^). WC, waist circumference; SBP, systolic blood pressure; DBP, diastolic blood pressure; BP, blood pressure; BP categories: normal BP (SBP < 130 and/or DBP < 85 mmHg), prehypertension (SBP 130 to 139 and/or DBP 85 to 89 mmHg), hypertension (SBP ≥ 140 and/or DBP ≥ 90 mmHg and/or with antihypertensive treatment); FPG, fasting plasma glucose; GGT, γ-glutamyl transpeptidase; AST, aspartate aminotransferase; ALT, alanine aminotransferase; HDL-C, high-density lipoprotein; LDL-C, low-density lipoprotein; TG, triglycerides; dyslipidemia (TG ≥ 150 and/or HDL ≥ 40 mg/dL in men, ≥46 mg/dL in women and/or with lipid-lowering treatment); MetS, metabolic syndrome according to the IDF; Framingham-REGICOR: low risk (<5%), moderate risk (5–9.9%), high risk (10–14.9%), severe risk (>15%). SCORE: low risk (<0.99%), moderate risk (1–4.99%), high risk (5–9.99%), severe risk (>10%).

**Table 2:** Anthropometric characteristics and biochemical parameters of the study population by FLI categories.

Variable	FLI < 60 (*n* = 41)	FLI ≥ 60 (*n* = 145)	*OR* (95% CI)	*p*-Value *
Age (years)	58.56 (10.96)	59.46 (10.16)	1.01 (0.97–1.04)	0.623
Social class				0.225
White collar	6 (14.6)	34 (23.4)	Ref.	
Blue collar	35 (85.4)	111 (76.6)	0.55 (0.21–1.41)	
Smoking status				
Never	22 (53.7)	62 (42.8)	Ref.	0.425
Former	13 (31.7)	61 (42.1)	1.61 (0.74–3.448)	
Current	6 (14.6)	22 (15.2)	1.60 (0.46–3.62)	
BMI (kg/m^2^)	28.62 (1.55)	33.33 (3.23)	2.58 (1.88–3.55)	<0.001
BMI categories				<0.001
Overweight	32 (78.0)	17 (11.7)	Ref.	
Obese	9 (22.0)	128 (88.3)	26.35 (10.75–64.57)	
WC (cm) in men	100.10 (5.61)	110.94 (8.44)	1.25 (1.11–1.41)	<0.001
WC (cm) in women	91.47 (6.50)	106.46 (8.32)	1.28 (1.15–1.42)	<0.001
SBP (mmHg)	130.51 (14.28)	134.97 (13.97)	1.02 (0.99–1.05)	0.075
DBP (mmHg)	81.40 (8.62)	84.44 (9.39)	1.03 (0.99–1.07)	0.065
BP categories				0.151
Normal	11 (26.8)	21 (14.5)	Ref.	
Prehypertension	6 (14.6)	19 (11.3)	1.57 (0.48–5.10)	
Hypertension	24 (58.5)	105 (72.4)	2.27 (0.96–5.33)	
FPG (mg/dL)	108.02 (5.91)	108.97 (6.34)	1.02 (0.96–1.08)	0.396
HbA1c ^∇^	5.88 (0.29)	5.89 (0.33)	1.10 (0.33–3.70)	0.801
GGT (IU/L)	25.32 (10.29)	49.43 (66.90)	1.06 (1.02–1.10)	<0.001
AST (IU/L) ^+^	19.68 (5.00)	25.43 (12.87)	1.11 (10.30–1.19)	<0.001
ALT (IU/L) °	19.09 (7.81)	30.17 (20.47)	1.10 (1.05–1.16)	<0.001
Cholesterol (mg/dL)	194.22 (31.18)	199.43 (36.03)	1.00 (0.99–1.01)	0.402
HDL-C (mg/dL) n= 184	53.85 (11.88) *n* = 40	48.90 (12.11) *n* = 144	0.96 (0.94–0.99)	0.023
LDL-C (mg/dL) n= 181	120.63 (28.37) *n* = 41	119.24 (30.22) *n* = 140	0.99 (0.98–1.01)	0.792
TG (mg/dL)	94.46 (27.70)	168.32 (157.88)	1.02 (1.01–1.04)	<0.001
Presence of dyslipidemia				
	21 (51.2)	86 (59.3)	1.39 (0.69–2.80)	0.355
Presence of MetS	20 (48.8)	116 (81.1)	5.91 (2.34–14.93)	<0.001
REGICOR	3.53 (2.27)	4.84 (2.73)	1.29 (1.07–1.56)	0.006
Categories of Framingham-REGICOR				0.005
Low risk ^a^	34 (82.9)	78 (54.5)	Ref.	
Moderate risk ^b^	6 (14.6)	55 (38.5)	3.99 (1.57–10.16)	
High risk	1 (2.4)	10 (7.0)	4.35 (0.53–35.40)	
SCORE	2.35 (2.38)	3.07 (2.67)	1.12 (0.96–1.31)	0.121
Categories of SCORE				0.172
Low risk	13 (31.7)	38 (26.6)	Ref.	
Moderate risk	24 (58.5)	72 (50.3)	1.02 (0.47–2.24)	
High risk	4 (9.8)	33 (23.1)	2.82 (0.83–9.50)	

Data are expressed as mean (standard deviation) or count (percentage). * *p*-values for comparison between FLI categories, obtained by independent sample *t*-test for continuous variables or by Chi-square test for categorical variables. Post hoc test by Bonferroni method: ^a^ significant difference between FLI ≥ 60 and FLI < 60, ^b^ no significant difference between FLI ≥ 60 and FLI < 60. ^+^AST available for *n* = 179 (FLI < 60 *n* = 41; FLI ≥ 60 *n* = 138); ° ALT available for *n* = 182 (FLI < 60 *n* = 41; FLI ≥ 60 *n* = 138); **^∇^** HbA1c available for *n* = 149 (FLI < 60 *n* = 31; FLI ≥ 60 *n* = 118). Abbreviations and category definition: FLI, fatty liver index; OR, odd ratios; 95% CI, confidence intervals; BMI, body mass index; BMI categories: overweight (BMI 25 to 30 kg/m^2^); obese (BMI ≥ 30 kg/m^2^). WC, waist circumference; SBP, systolic blood pressure; DBP, diastolic blood pressure; BP, blood pressure; BP categories: normal BP (SBP < 130 and/or DBP < 85 mmHg), prehypertension (SBP 130 to 139 and/or DBP 85 to 89 mmHg), hypertension (SBP ≥ 140 and/or DBP ≥ 90 mmHg and/or with antihypertensive treatment); FPG, fasting plasma glucose; GGT, γ-glutamyl transpeptidase; AST, aspartate aminotransferase; ALT, alanine aminotransferase; HDL, high-density lipoprotein; LDL, low-density lipoprotein; TG, triglycerides; dyslipidemia (TG ≥ 150 and/or HDL ≥ 40mg/dL in men, ≥46 mg/dL in women and/or with lipid-lowering treatment); MetS, metabolic syndrome according to the IDF; Framingham-REGICOR: low risk (<5%), moderate risk (5–9.9%), high risk (10–14.9%), severe risk (>15%). SCORE: low risk (<0.99%), moderate risk (1–4.99%), high risk (5–9.99%), severe risk (>10%).

## Data Availability

The data presented in this study are available in article.
